# Effect of maternal separation on mitochondrial function and role of exercise in a rat model of Parkinson’s disease

**DOI:** 10.1007/s11011-012-9305-y

**Published:** 2012-04-18

**Authors:** Sharief Hendricks, Edward Ojuka, Lauriston A. Kellaway, Musa V. Mabandla, Vivienne A. Russell

**Affiliations:** 1 MRC/UCT Exercise Science and Sports Medicine Research Unit, Department of Human Biology, Faculty of Health Sciences, University of Cape Town, Cape Town, South Africa; 2 School of Laboratory Medicine and Medical Sciences, College of Health Sciences, University of KwaZulu-Natal, Durban, 4000 South Africa

**Keywords:** Developmental stress, 6-OHDA, Dopamine, Striatum, Prefrontal cortex

## Abstract

Early life stress, such as maternal separation, causes adaptive changes in neural mechanisms that have adverse effects on the neuroplasticity of the brain in adulthood. As a consequence, children who are exposed to stress during development may be predisposed to neurodegenerative disorders in adulthood. A possible mechanism for increased vulnerability to neurodegeneration may be dysfunctional mitochondria. Protection from neurotoxins, such as 6-hydroxydopamine (6-OHDA), has been observed following voluntary exercise. The mechanism of this neuroprotection is not understood and mitochondria may play a role. The purpose of this study was to determine the effects of maternal separation and exercise on mitochondrial function in a rat model of Parkinson’s disease. Maternally separated (pups separated from the dam for 3 h per day from postnatal day (P) 2–14) and non-separated rats were placed in individual cages with or without attached running wheels for 1 week prior to unilateral infusion of 6-OHDA (5 μg/4 μl, 0.5 μl/min) into the left medial forebrain bundle at P60. After 2 h recovery, rats were returned to their cages and wheel revolutions recorded for a further 2 weeks. On P72, the rats’ motor function was assessed using the forelimb akinesia test. On P74, rats were sacrificed for measurement of mitochondrial function. Exercise increased the respiratory control index (RCI) in the non-lesioned hemisphere of 6-OHDA-lesioned rats. This effect was evident in the striatum of non-separated rats and the prefrontal cortex of maternally separated rats. These results suggest that early life stress may reduce the adaptive response to exercise in the striatum, a major target of dopamine neurons, but not the prefrontal cortex in this model of Parkinson’s disease.

## Introduction

Stress challenges the homeostatic state of an organism resulting in a cascade of adaptive physiological responses in an attempt to maintain homeostasis (Charmandari et al. 2005). These physiological responses include alterations in neuroendocrine, autonomic and immune function (Carpenter et al. 2007; Levine 2005). The magnitude of these physiological responses is determined by the nature and duration of the stressor. If a stressful experience endures for a prolonged period, the ability of the organism to adapt may fail resulting in increased activation of the hypothalamic-pituitary-adrenal (HPA) axis (Plotsky and Meaney 1993), leading to disorders such as anxiety and depression later in life (Heim et al. 2001). These adverse effects of prolonged exposure to a stressor are exacerbated in infants since their adaptive response mechanisms are not fully developed (Meaney et al*.* 1989). One such stress during infancy is extended separation from the mother.

Studies have shown that maternal separation may increase the response of the adrenal gland and increase fearfulness in adulthood (Faure et al*.* 2006; Schmidt et al*.* 2004). The mother-infant relationship is regulated by a set of physical attributions such as touch, warmth, licking, odour and milk letdown (Hofer 1971). With maternal separation, all these regulatory components are simultaneously lost and, over time, may alter the infant’s behavioural, neuroendocrine and autonomic systems and can shape behaviour and development of the infant. The effects of depriving rat pups of maternal care have been the focus of many studies which have shown that experiences early in life alter their ability to respond to a perceived stressor in adulthood (Faure et al*.* 2006; Heim et al*.* 2001). Rat pups separated from their mothers for 3 h per day from postnatal day (P) 2 to 14, and subsequently exposed to a stressor, had significantly decreased basal plasma ACTH and corticosterone levels compared to controls (Faure et al*.* 2006). Furthermore, they were characterised by a decrease in locomotor activity and increased anxiety- or fear-like behaviour compared to controls (Kalinichev et al*.* 2002; Lee et al*.* 2007). These adverse effects of maternal separation seem to be modulated by factors such as the age of the pup, the duration of the separation and also the strain and gender of the rats (Eklund and Arborelius 2006; Slotten et al*.* 2006; Sterley et al*.* 2011).

A possible mechanism for these physiological responses to stress is impaired functioning of the mitochondria. It has been reported that chronic stress causes an over-production of nitric oxide which could inhibit mitochondrial respiratory chain function and give rise to oxidative stress (Madrigal et al*.* 2001). Moreover, excessive production of free radicals by the mitochondria, and the inability of neurons to neutralize them may lead to mitochondrial dysfunction and neuronal death (Beal 1996). Dysfunctional mitochondria have been associated with the pathogenesis of several neurodegenerative diseases such as Parkinson’s disease, Alzheimer’s disease, Friedreich’s ataxia, multiple sclerosis, amyotrophic lateral sclerosis, Huntington’s disease and diabetes (Madrigal et al*.* 2001). Rat models of Parkinson’s disease which use the neurotoxin, 6-hydroxydopamine (6-OHDA), to lesion dopamine neurons in the brain, have provided evidence that autoxidation of 6-OHDA elicits neurodegeneration by producing reactive oxygen species that interfere with mitochondrial processes (Kulich et al*.* 2007).

There is evidence to suggest that dopamine neurons in the substantia nigra are spared when rats are forced to exercise the impaired limb after 6-OHDA injection into the medial forebrain bundle (Tillerson et al*.* 2001). Furthermore, neuroprotection from the toxic effects of 6-OHDA has also been observed following voluntary exercise (Mabandla et al*.* 2004). Despite these findings, the mechanism of neuroprotection is not entirely clear (Cotman et al*.* 2007; Mabandla et al*.* 2004; Tillerson et al*.* 2003). A possible mechanism for neuroprotection may be through the mitochondria. Studies have found that exercise up-regulates proteins involved in mitochondrial function while decreasing proteins related to oxidative stress (Navarro et al*.* 2004). The purpose of this study was to determine the effects of maternal separation and exercise on mitochondrial function in a rat model of Parkinson’s disease.

## Methods

### Animals

Thirty-nine male Sprague–Dawley rats (Rattus Norvegicus) weighing between 180 g and 280 g were used in the study. Rats were housed in a temperature-controlled room (21–24°C) in the Satellite Animal Facility at the University of Cape Town. Animals had access to standard rat chow and water *ad libitum*. The housing facility was maintained on a 12-hour light/dark cycle (lights on at 06 h00). The study was approved by the Faculty of Health Sciences Animal Research Ethics Committee of the University of Cape Town and adhered to international guidelines. On postnatal day 1 (P1), rats were sexed and culled to 8 males per litter. If the litter had less than 8 males, female littermates were added to standardize litter size and to allow for equal suckling from the dam.

### Maternal separation

Maternal separation was performed as described by Daniels et al*.* (2004). For the experimental group the mother was physically removed from the home cage and placed in a separate clean plexiglass cage with clean bedding from P2 to P14. A small amount of bedding from the home cage was placed in the holding cage to decrease the stress the dam might be exposed to due to the separation. The pups were taken to a different room (separation room) to prevent communication with the dam (by means of ultrasound vocalization) for a period of 3 h from 09 h00 to 12 h00 daily. The temperature in the separation room was maintained between 31 and 34°C to prevent hypothermia. After the 3-hour separation period the pups were returned to the Satellite Animal Facility and reunited with their dams. The home cage was cleaned every fourth day and great care was taken not to physically disturb the pups and to minimize other factors (smell, sound) that could cause stress to the rat pups. During the cleaning of the home cages, the dam was removed and about half of the bedding which was not occupied by the pups was changed to ensure that the dam recognised the odour of her cage and her litter when returned to her home cage. The non-separated pups were kept with their dams in their home cages, under normal housing conditions in the Satellite Animal Facility. After P14 the litters were left with their dams until P21 when the litters were weaned.

### Voluntary exercise

From P21 to P48 the rats were reared under normal housing conditions (standard rat chow and water *ad libitum*). On P48 rats were transferred to a room with a 23 h00-11 h00 light/dark cycle in order to facilitate recording and observation of their activity. After 6 days of adaptation to the new light/dark cycle, on P54, 20 maternally separated rats and 19 non-maternally separated rats were weighed and further divided into exercised (runners) and non-exercised (non-runners) groups. Ten maternally separated (MS) rats, and 9 non-separated (NS) rats were placed in cages with attached running wheels with a counter device that recorded the number of revolutions. One complete revolution measured one meter in distance. The remaining maternally separated rats (MS non-runners, *n* = 10) and non-separated rats (NS non-runners, *n* = 10) were placed in plexiglass cages that allowed 2 rats to be housed separately using a divider. The number of revolutions the rats made in the running wheels was recorded daily between 10 h00 and 11 h00 prior to the onset of their dark cycle.

### Stereotaxic surgery

Stereotaxic surgery was performed according to Mabandla et al*.* (2004). On P60, the rats were weighed and taken to the surgery room. A norepinephrine transporter blocker, desipramine (15 mg/kg, Sigma St. Louis, MO, U.S.A), was injected intraperitoneally 30 min before surgery to prevent uptake of the neurotoxin, 6-OHDA, by noradrenergic neurons. Rats were deeply anaesthetised with a mixture of halothane and oxygen administered by means of a calibrated Blease Vaporiser (DATUM). All rats received 6-OHDA.HCl (5 μg/4 μl saline, Sigma, St. Louis, MO, U.S.A) infusion unilaterally (0.4 μl/min) into the left medial forebrain bundle at the following stereotaxic co-ordinates: 4.7 mm anterior to lambda, 1.6 mm lateral to midline and 8.4 mm ventral to dura, according to the rat brain atlas of Paxinos and Watson (1986). The infusion needle remained in the medial forebrain bundle for a period of 4 min post infusion to allow the neurotoxin to disperse into the tissue. Following gentle retraction of the needle, the burr-hole was sealed with sterile bone-wax and the scalp incision was swabbed with betadine/alcohol solution and sutured. The rats were allowed to recover from anaesthesia before they were returned to their respective cages. The number of revolutions made by the rats in cages with attached running wheels was recorded for a further 14 days after the surgery.

### Behavioural tests

On P72 (twelve days post lesion), the rats were assessed for motor function deficit. The rats were taken from the Satellite Facility at least 1 h before testing so as to enable the rats to acclimatize to the new conditions in the behavioural testing room. The light intensity in the behavioural testing room was 48 lux.

#### Forelimb akinesia test (step test)

The step test provides a robust measure of impairment in movement initiation, and thus measures the severity of the effect of the 6-OHDA lesion on limb function (Tillerson et al*.* 2001). The rat was supported by its torso, such that the hindquarters and the forelimb not being tested were held above the testing surface, allowing the rat to transfer its bodyweight onto the forelimb being tested. With the use of the thumb and index finger, the forelimb not being tested and head were held firmly to minimize head movement and to simultaneously gently propel the rat forward. The length of step of each forelimb was recorded in 3 successive trials and the mean step length was calculated.

On P74, rats were sacrificed and their brains rapidly removed from the skull for measurement of mitochondrial function using an Oxygraph oxygen electrode (Hansatech Instruments, UK).

### Mitochondrial function (Oxygen Consumption)

Left and right prefrontal cortex and striatum were rapidly dissected from the brain, on an ice-cold glass plate, weighed and homogenized in 20 mM phosphate buffer (20 mM potassium phosphate, 20 mM potassium chloride, 1.6 mM EDTA, 5 mM magnesium chloride, 1 mM sodium malate, 10 mM sodium pyruvate 123 mM sucrose, 2 mM Tris, pH 7.4) and oxygen consumption measured in an Oxygraph, as an index of mitochondrial function. Two ml of homogenate was placed in the Oxygraph chamber and allowed to equilibrate to 39°C. Basal respiration was recorded for 5–10 s prior to addition of 1 mM adenosine diphosphate (ADP) to the chamber and oxygen tension was recorded until depletion. Respiratory control index (RCI) which indicates the tightness of coupling between respiration and phosphorylation, was calculated from the rate of oxygen consumption during ADP oxidation to ATP relative to the rate of oxygen consumption after ADP depletion. The phosphate:oxygen ratio (P:O) was also calculated from the oxygen consumption data. The P:O ratio provides a measure of the relationship between ATP synthesised and oxygen consumed. The validity of the technique was demonstrated by addition of NADH to the brain tissue homogenate prior to the measurement of oxygen consumption which did not alter the rate of respiration, thus confirming that the mitochondria were intact. The rate of oxygen consumption was not influenced by aeration of the homogenate, indicating that sufficient oxygen was present in the sample to measure mitochondrial respiration. The mean RCI was 10.8 which compares favourably with reported values of 2.2–4.9 for isolated brain mitochondria (Sugiyama and Fujita 1985; Xiong et al. 1999; Costa and Colleoni 2000).

### Protein determination

Protein concentration was determined according to the method described by Bradford (1976). Briefly, the Bradford protein assay is a colorimetric assay. A series of protein standards were prepared (0–1500 μg BSA/ml). Serial dilutions of the sample to be measured were also prepared. Each sample (standard and unknown) was pipetted into a spectrophotometer tube before adding a dye for the color reaction. The absorbance was measured using a spectrophotometer. The absorbance of the standards versus their concentration was plotted on graph paper following which the concentration of the unknown sample was extrapolated.

### Statistical analysis

Statistica (Statsoft Inc., Oklahoma, USA) was used for statistical analysis of the data. The t-test for dependent variables was used to determine differences between lesioned and non-lesioned hemispheres. Two-way analysis of variance (ANOVA) was performed to detect significant differences between treatment groups. Significant difference was accepted at *p* < 0.05. When the ANOVA showed a significant difference, the Newman-Keuls post-hoc test was used. Results are reported as mean ± standard error of the mean (mean ± SEM).

## Results

Rats that had access to running wheels are referred to as runners and those that were in plexiglass cages without running wheels as non-runners. There was no difference between maternally separated rats and non-separated rats in the distance covered. The average distance increased from 847 ± 197 and 669 ± 243 m/day, respectively to an average of 4400 m/day after 17 days.

### Forelimb akinesia test

**Fig. 1:**
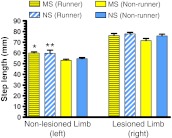
Mean step length of lesioned (right) and non-lesioned (left) forelimb of maternally separated (MS) and non-maternally separated (NS) runners and non-runners. *Non-lesioned limb of maternally separated (MS) runners vs non-runners, (*p* < 0.05). **Non-lesioned limb of non-separated (NS) runners vs non-separated (NS) non-runners, (*p* < 0.05) and maternally separated (MS) non-runners (*p* < 0.05). Results are the mean ± SEM

The mean step length taken by the unimpaired limb (left limb, ipsilateral to the lesioned hemisphere) was significantly shorter than the mean step length taken by the impaired limb in all 6-OHDA lesioned rats (right limb, t-test for dependent variables, *p* < 0.0001, Fig. 1). Two-way ANOVA revealed that maternal separation did not have an effect on the mean step length taken by the impaired limbs, while exercise significantly increased the mean step length of the unimpaired limb (F_(2,35)_ = 6.7, *p* < 0.0.01). Exercise resulted in the mean step length taken by the unimpaired limb (left) of the non-separated (NS) runners being significantly greater than the mean step length of the non-separated (NS) non-runners and maternally separated (MS) non-runners (*p* < 0.05). Also, the step length of the unimpaired limb (left) of the maternally separated (MS) runners was significantly greater than that of the maternally separated (MS) non-runners (*p* < 0.05).

### Oxygen consumption

There were no differences between the lesioned and non-lesioned hemispheres in terms of the P:O ratio and RCI in the striatum and prefrontal cortex of 6-OHDA-lesioned rats.

Two-way ANOVA revealed that exercise and maternal separation did not affect the P:O ratio, or ADP-stimulated oxygen consumption, while exercise increased the RCI in both striatum and prefrontal cortex in both hemispheres, by reducing the ADP-depleted oxygen consumption. This was evidenced by a significant interaction between maternal separation and exercise in striatal ADP-depleted oxygen consumption in both lesioned (F_(1,36)_ = 4.95, *p* < 0.05) and non-lesioned hemispheres (F_(1,36)_ = 6.02, *p* < 0.05). Exercise reduced ADP-depleted oxygen consumption in the striatum of non-separated rats but not in maternally separated rats. There was a significant effect of exercise on ADP-depleted oxygen consumption in the prefrontal cortex of both lesioned (F_(1,36)_ = 15.6, *p* < 0.0005) and non-lesioned hemispheres (F_(1,36)_ = 19.5, *p* < 0.0001). Exercise reduced ADP-depleted oxygen consumption in the prefrontal cortex in the lesioned (*p* < 0.005) and non-lesioned hemisphere (*p* < 0.05) of the non-separated rats and in the non-lesioned hemisphere of maternally separated rats (*p* < 0.005).

**Fig. 2:**
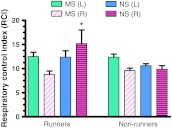
Striatal Respiratory Control Index (RCI) for lesioned (L) and non-lesioned (R) hemispheres of maternally separated (MS) and non-maternally separated (NS) runners and non-runners. * Non-lesioned hemisphere (R) of non-separated (NS) runners significantly greater than maternally separated (MS) runners and non-runners and non-separated (NS) non-runners, (*p* < 0.05). Results are the mean ± SEM

In the striatum, maternal separation significantly affected the RCI (F_(1,36)_ = 4.87, *p* < 0.05) and there was a significant stress*exercise interaction (F_(1,36)_ = 4.22, *p* < 0.05). Exercise increased the RCI in non-separated rats but not in rats that had been maternally separated (Fig. 2). Post-hoc Newman Keuls test revealed that the RCI of the striatum in the non-lesioned hemisphere (R) of non-separated (NS) runners was significantly greater than that of the non-separated (NS) non-runners and maternally separated (MS) runners and non-runners (*p* < 0.05, Fig. 2).

**Fig. 3:**
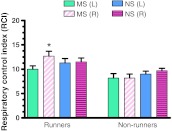
Prefrontal cortex Respiratory Control Index (RCI) of lesioned (L) and non-lesioned (R) hemispheres of maternally separated (MS) and non-maternally separated (NS) runners and non-runners. * Non-lesioned hemisphere (R) of maternally separated (MS) runners significantly greater than non- runners, (*p* < 0.05). Results are the mean ± SEM

Two-way ANOVA revealed that exercise increased the RCI in the prefrontal cortex in both lesioned (F_(1,36)_ = 7.00, *p* < 0.02) and non-lesioned hemispheres (F_(1,36)_ = 15.69, *p* < 0.0005). Post-hoc Newman Keuls test revealed that the RCI of the prefrontal cortex in the non-lesioned hemisphere (R) of the maternally separated (MS) runners was significantly greater than that of the maternally separated (MS) non-runners (*p* < 0.05, Fig. 3).

## Discussion

The step test is used to monitor forelimb akinesia in the affected limb as a useful behavioural marker of unilateral 6-OHDA lesion of dopamine neurons in the rat model of Parkinson’s disease. The significant difference between affected limb and non- affected limb in the control group (non-separated non-runners) is a clear indication of the effect of the 6-OHDA as a consequence of dopamine neuron loss on the injected side. In agreement with previous findings (Mabandla and Russell 2010), the 6-OHDA lesion impaired movement initiation in the contralateral (right) forelimb and exercise increased the step length of the unimpaired limb. This may be the result of a compensatory mechanism to minimize motor imbalance due to differences in forelimb step length. Similar to previous findings (Mabandla and Russell 2010), exercise did not affect the impaired limb of maternally separated rats. However, in contrast, the step length of the impaired limb of non-separated runners was not reduced by exercise. This could not be attributed to the age of the rats or the amount of exercise, since these were similar across the studies. The distance covered by the rats in the cages with attached running wheels increased similarly over time, to reach a plateau of about 4400 m/day which is in agreement with Mabandla and Russell (2010). The only difference is the degree of asymmetry which was much smaller in the present study than in the Mabandla and Russell (2010) study which may have provided greater sensitivity to the effects of exercise.

Mitochondrial function was not altered by the 6-OHDA lesion. There were no differences between the lesioned and non-lesioned hemispheres in terms of the P:O ratio or the RCI in the striatum and prefrontal cortex of 6-OHDA-lesioned rats. Exercise did not affect the P:O ratio but the RCI was increased by exercise in striatal tissue in the non-lesioned hemisphere of non-separated rats. Since there was no difference between groups in the ADP-stimulated consumption of oxygen, the exercise-induced increase in the RCI appeared to be due to a decrease in ADP-depleted respiration in the non-separated runners. These findings suggest that exercise decreased oxygen consumption by non-mitochondrial mechanisms e.g. free radicals, possibly by up-regulation of superoxide dismutase (Somani et al. 1995; Aksu et al. 2009; Daniels et al. 2011), in the non-lesioned hemisphere of 6-OHDA-lesioned rats and that maternal separation blocked this adaptive response to exercise in the striatum.

In the prefrontal cortex, exercise similarly increased the RCI, particularly in the non-lesioned hemisphere of maternally separated rats. It therefore appeared that maternal separation did not impair the beneficial effect of exercise on prefrontal cortex function. These findings are consistent with reports that voluntary exercise decreases superoxide radicals resulting from metabolism of 6-OHDA, by increasing expression of superoxide dismutase in rat brain tissue (Somani et al. 1995; Aksu et al. 2009; Daniels et al. 2011). Exercise either decreased or did not affect glutathione peroxidase levels (Somani et al. 1995; Aksu et al. 2009) and decreased peroxiredoxin-6 levels in rat hippocampus (Daniels et al. 2011). Peroxiredoxin-6 has the unique capacity to reduce membrane phospholipid hydroperoxides (Trudel et al. 2009) thereby reducing free radical damage which could account for the increased RCI in brain tissue of exercised rats.
